# Impact of San Francisco’s Toy Ordinance on Restaurants and Children’s Food Purchases, 2011–2012

**DOI:** 10.5888/pcd11.140026

**Published:** 2014-07-17

**Authors:** Jennifer J. Otten, Brian E. Saelens, Kristopher I. Kapphahn, Eric B. Hekler, Matthew P. Buman, Benjamin A. Goldstein, Rebecca A. Krukowski, Laura S. O’Donohue, Christopher D. Gardner, Abby C. King

**Affiliations:** Author Affiliations: Brian E. Saelens, University of Washington and Seattle Children’s Research Institute, Seattle, Washington; Kristopher I. Kapphahn, Benjamin A. Goldstein, Laura S. O’Donohue, Christopher D. Gardner, Abby C. King, Stanford University School of Medicine, Stanford, California; Eric B. Hekler, Matthew P. Buman, School of Nutrition and Health Promotion, Arizona State University, Phoenix, Arizona; Rebecca A. Krukowski, University of Tennessee Health Science Center, Memphis, Tennessee.

## Abstract

**Introduction:**

In 2011, San Francisco passed the first citywide ordinance to improve the nutritional standards of children’s meals sold at restaurants by preventing the giving away of free toys or other incentives with meals unless nutritional criteria were met. This study examined the impact of the Healthy Food Incentives Ordinance at ordinance-affected restaurants on restaurant response (eg, toy-distribution practices, change in children’s menus), and the energy and nutrient content of all orders and children’s-meal–only orders purchased for children aged 0 through 12 years.

**Methods:**

Restaurant responses were examined from January 2010 through March 2012. Parent–caregiver/child dyads (n = 762) who were restaurant customers were surveyed at 2 points before and 1 seasonally matched point after ordinance enactment at Chain A and B restaurants (n = 30) in 2011 and 2012.

**Results:**

Both restaurant chains responded to the ordinance by selling toys separately from children’s meals, but neither changed their menus to meet ordinance-specified nutrition criteria. Among children for whom children’s meals were purchased, significant decreases in kilocalories, sodium, and fat per order were likely due to changes in children’s side dishes and beverages at Chain A.

**Conclusion:**

Although the changes at Chain A did not appear to be directly in response to the ordinance, the transition to a more healthful beverage and default side dish was consistent with the intent of the ordinance. Study results underscore the importance of policy wording, support the concept that more healthful defaults may be a powerful approach for improving dietary intake, and suggest that public policies may contribute to positive restaurant changes.

## Introduction

The marketing of unhealthful foods and beverages through toys and other incentives contributes to the development of unhealthy eating patterns and behaviors that lead to child obesity (1,2). In 2009, 10 of the top fast food restaurant chains spent $341 million to acquire toys to distribute with children’s meals (a combination of entrée, side dish, and beverage that is targeted to children through marketing or age restrictions) (3). During the same year, these restaurants sold slightly more than 1 billion meals with toys to children aged 0 through 12 (3). A study evaluating the content of children’s meals at the top 50 restaurant chains found that 97% of children’s meal combinations did not meet evidence-based child nutrition standards and that 56% did not have a single children’s meal meeting these standards (4).

Public health leaders have called for policy-driven strategies aimed at improving food environments and marketing practices (1,5,6). On December 1, 2011, San Francisco, California, became the first US city to enact an ordinance prohibiting restaurants from giving away free toys or other incentives with children’s meals or with foods and beverages not meeting minimal nutritional criteria (Box) (7). The intent of the ordinance was “to improve the health of children and adolescents in San Francisco by setting healthy nutritional standards for children’s meals sold at restaurants in combination with free toys or other incentive items” (8).

**Box. Summary of the Healthy Food Incentives Ordinance (No. 290–10), San Francisco, California.**

The San Francisco County ordinance prohibits restaurants in the county from giving away free toys or other incentive items, such as games, trading cards, admission tickets, or other consumer products —whether physical or digital — with children’s meals (ie, any combination of food items offered together for a single price) that exceed the following criteria for each meal:

• 600 calories

• 35% of total calories from fat, 10% of total calories from saturated fat (with some exceptions for sources of total and saturated fat), and/or 0.5 grams of trans fat

• 640 mg of sodium

In addition, the meal must include at least 0.5 cups of fruit and 0.75 cups of vegetables (unless breakfast, which must include at least 0.5 cups of fruit or 0.5 cups of vegetables)

Single food and beverage items must not exceed

• 35% of total calories from fat

• 10% of total calories from added sweeteners

The ordinance allows free toys or other incentives with foods, beverages, and meals that meet these criteria. The criteria were based on nutrient recommendations and standards for children provided by the Institute of Medicine of The National Academies and the US National School Lunch Standards (K–6) (9,10).

Few ordinances of this type have been enacted or evaluated. The first US county-level ordinance of this type was implemented in Santa Clara County (no. NS-300–820) (11). Although this ordinance applied to only a few restaurants in unincorporated areas of the county, research suggested it improved restaurant food environments and practices (12). Another study found that children were significantly more likely to choose a more healthful meal among an array of healthful and less healthful meals when only more healthful meals were offered with a toy (13).

The objective of this evaluation was to examine the impact of the San Francisco Healthy Food Incentives Ordinance at ordinance-affected fast food chain restaurants on restaurant response and the energy and nutrient content of all orders and children’s-meal–only orders purchased for children.

## Methods

### Study design

The San Francisco Healthy Food Incentives Ordinance (also referred to as the “toy ordinance”) was adopted on November 23, 2010, and enacted on December 1, 2011. Our study used a pre–post design to evaluate this ordinance, and data were collected on local and national restaurant responses (eg, marketing environment, menu changes) and from caregiver/child dyads who were interviewed at two points before ordinance enactment and at one point after enactment. Pre-ordinance time point 1 was from January through March 2011, immediately after adoption and 9 to 12 months before enactment; pre-ordinance time point 2 was from October through November 2011, one or two months before enactment; and post-ordinance was from January through March 2012, one to three months after enactment and a seasonal match with pre-ordinance time point 1.

### Study setting and population

The ordinance applied to all restaurants in San Francisco that sold children’s meals with toys or other incentives (n = 98). We narrowed the sample to 2 global restaurant chains (Chain A [n = 19]; Chain B [n = 11]) that operated in San Francisco during the study period. This decision was based on budget constraints and research (12,14) showing that caregiver/child dyads felt that toys affected purchases most strongly at major restaurant chains, a finding supported by research showing that these two chains market toys with children’s meals to children more frequently than do other chains (15). We then excluded two Chain A restaurants because of lack of public space in which to conduct the street-intercept surveys.

In 2009, San Francisco was the 12th largest city (population, ~1 million); the population was 58% white, 31% Asian, 7% black; and 14% Hispanic (16). In 2010, 32% of children in San Francisco were obese or overweight (17). Our target study population was parents or caregivers with children (aged 0–12 y) who were customers of the restaurants.

### Data collection

**Changes in children’s meals at the local and national level.** Because there were many ways a restaurant could comply with the ordinance and it was unknown how restaurants would comply, we tracked local changes in menus and restaurant practices by reviewing local press releases, by direct observation, and by test purchases by trained staff. We tracked menus and restaurant practices at the national level by reviewing items in the news media and press releases, announcements, and menus posted on corporate websites. All children’s meal combinations (each combination of entrée, side dish, and beverage) were evaluated for nutritional content and whether they met ordinance criteria by using information from corporate websites.

**Street-intercept survey.** During 8 weekends of each of the three study time points, a team of 4 to 6 trained research personnel collected data during the lunch hour (generally 11:00 am to 3:00 pm) through street-intercept surveys of parent–caregiver/child dyads. Data collection occurred only on weekends to reduce the potential effect of school being in or out of session.

Research personnel were randomly assigned a set of restaurants and instructed to collect data at each location until they collected 10 surveys or 5 hours of data, whichever occurred first; restaurants where 10 surveys had been collected in less than 5 hours were revisited until 5 hours of data were accumulated.

We used a street-intercept survey method to invite parents or caregivers to participate in the study, a method used in studies on menu-labeling legislation (18,19). Parents or caregivers with children were approached as they entered the restaurant on foot and were offered a $5 restaurant gift card to complete a 10- to15-minute interviewer-administered survey after they completed their purchases. Participants were informed that the research topic was child food purchases and were asked to provide their receipt; no mention was made of the ordinance at interview initiation. The study protocol was approved by the Stanford University institutional review board; parents and caregivers provided verbal consent.

The street-intercept survey collected data on

- Eating-out habits and purchase priorities (eg, frequency of fast food visits by month, reason for choosing this restaurant);
- Habits, priorities, and requests related to children’s meals and toys (eg, frequency a children’s meal is chosen, importance of and request for toys);
- Awareness of the toy ordinance and calorie labels; and
- Foods and beverages ordered and demographic and anthropometric data (ie, birthdate, sex, race/ethnicity, and height and weight) for both parent/caregiver and child.

We collected surveys from 863 dyads at three time points (pre-ordinance 1 [n = 296]; pre ordinance 2 [n = 286]; post-ordinance [n = 281]). We excluded 101 surveys for the following reasons: survey was missing information on food orders or restaurant location (n = 58), food orders could not be verified (n = 14), or the children surveyed were older than 12, the parent or caregiver was younger than 18, or data on age were missing (n = 29). The final survey sample consisted of 762 dyads.

### Nutritional analysis of food and beverage purchases

A nutritional database was created for menu items available during each time point using data provided by corporate websites. For special or limited-time items, data were often unavailable. When data were unavailable, items were verified for existence, and nutritional information was often found in an announcement or press release. If an item could not be verified or adequate nutrition information found, we excluded the survey that mentioned the item. If the size of an item was not clear, we assumed it was the smallest size available. If details of a customization (eg, extra cheese, sauce, salad dressing, or beverage type) were not clear, we used average energy and nutrient values for all customization items in the same category. The following variables were calculated for each item purchased and aggregated by order per person, including customizations: calories, fat, saturated fat, trans fat, sodium, sugar, fiber, and protein. As an a priori decision, we based data for orders on self-report from parents or caregivers rather than receipts because our pilot work at Santa Clara County fast food restaurants found that receipts were incorrect (eg, inaccurate sandwich types) or contained insufficient information (eg, type of sauce or beverage) in 50% of test purchases conducted by trained staff.

### Statistical analysis

All analyses were conducted with R 3.1 [R Foundation for Statistical Computing, Vienna, Austria]. The main outcomes of interest were differences across time in mean energy content (kcal) per purchase for all children surveyed and for children ordering only children’s meals. Outliers were verified for plausibility and validity by cross-checking with surveys; all were plausible and valid. Because energy data were skewed, we used a nonparametric Kruskal–Wallis test to analyze the main outcomes overall and stratified by race/ethnicity, restaurant, and meal components (ie, entrée, side dish, and beverage). Multivariable regression analyses were performed but did not change inferences. We used Mann–Whitney *U* tests to test comparisons between time points, descriptive statistics to characterize study participants and survey responses, and χ^2^ tests to test for differences in categorical variables across time points.

## Results

Both restaurant chains responded to enactment of the toy ordinance by using the same compliance strategy: offering toys for an additional 10 cents with the purchase of a children’s meal. When restaurants began doing so, 88% of those in our sample purchasing children’s meals purchased the toy.

### Changes in children’s meals

No children’s meals at either chain met the ordinance nutritional criteria at any time during the study. However, changes at both chains during the study period affected the nutritional content of children’s meals.

**Chain A.** Beginning in September 2011 (after pre-ordinance time point 1 but before pre-ordinance time point 2) and only in California markets (20), Chain A did the following: one, revised the default side dish of its smaller child’s meal from 2.4 ounces of french fries and no fruit to 1.1 ounces of french fries and 1.2 ounces of apple slices (a reduction of 110 kcal, 6 g fat, and 130 mg sodium); two, revised the default side dish of its larger child’s meal from 2.4 ounces of french fries and no fruit to 2.4 ounces of french fries and 1.2 ounces of apple slices (an addition of 15 kcal); and three, stopped serving caramel sauce with its apple slices (a reduction of 70 kcal, 0.5 g fat, 9 g sugar, and 35 mg sodium). In addition, Chain A started offering fat-free chocolate milk in lieu of 1% chocolate milk (as a beverage option, not a default; a reduction of 40 kcal and 3 g fat and addition of 20 mg sodium) (20). These changes were confirmed through direct observation or test purchases. It was unclear whether the new default side dishes affected consumer purchases. Post-hoc analyses examining the proportion who received the default side dish among those ordering children’s meals showed a decrease from pre-ordinance time point 1 to pre-ordinance time point 2 coincident with Chain A menu changes (from 89% to 67%, *P* = .003), although no difference was found between seasonally matched pre-ordinance time point 1 and post-ordinance (from 89% to 83%, *P* = .49). (20).

**Chain B. **On July 31, 2011 (after pre-ordinance time point1 but before pre-ordinance time point 2), Chain B announced it would join the National Restaurant Association’s Kids LiveWell program (21). As part of the program, Chain B announced that soda and french fries would no longer be default in children’s meals but that employees would verbally offer all beverage and side dish options (22). In test purchases during pre-ordinance time point 2, alternative beverages and side dishes were not offered and apple slices with caramel continued to be sold for an extra cost (which varied by location) compared with french fries. However, in post-ordinance test purchases, Chain B employees offered beverage and side alternatives for children’s meals. We also observed at post-ordinance and validated by reviewing corporate menus that Chain B stopped serving caramel sauce with its children’s apple slices (a reduction of 45 kcal, 1g fat, 5g sugar, and 35 mg sodium).

### Street-intercept surveys

We found no significant demographic differences across time points, except for race/ethnicity (Table 1). Because we found no significant difference in calories per order by race/ethnicity between or among time points, we did not control for race/ethnicity in the primary analyses. Significantly fewer children’s meals were purchased at pre-ordinance time point 2 than at pre-ordinance time point 1 or at post-ordinance (*P* = .01), but the number of children meals purchased during the seasonally matched time points were not different (*P* = .79).

**Table 1:** Characteristics of Survey Sample (N = 762) Before and After Enactment^a^ of Toy Ordinance in Fast Food Restaurants, San Francisco, 2011–2012

Characteristic	Pre-Ordinance Time Point 1 (Jan–Mar 2011)	Pre-Ordinance Time Point 2 (Oct–Nov 2011)	Post-Ordinance Time Point (Jan–Mar 2012)	*P* Value^b^
**Surveys collected by restaurant chain, n**				
Chain A (n = 19 restaurants)	165	164	176	.73
Chain B (n = 11 restaurants)	91	82	84	
**Child’s age, n (%), y**				
0–2	29 (11.3)	14 (5.7)	15 (5.8)	.03
3–8	169 (66.0)	166 (67.5)	181 (69.6)	
9–12	58 (22.7)	66 (26.8)	64 (24.6)	
**BMI category for children aged ≥3 y^c^ (n = 450)^d^, n (%)**				
Underweight (<5th percentile)	7 (5.3)	9 (5.1)	10 (7.2)	.85
Normal (5th–85th percentile)	50 (37.6)	63 (35.4)	54 (38.8)	
Overweight or obese (≥85 percentile)	76 (57.1)	106 (59.6)	75 (54.0)	
**Child’s sex (n = 742)^d^, n (%)**				
Female	120 (48.2)	129 (53.8)	119 (47.0)	.28
**Child’s race/ethnicity (n = 720)^d^, n (%)**				
White	46 (20.6)	34 (14.1)	47 (18.4)	<.001
Black	28 (12.6)	29 (12.0)	31 (12.1)	
Asian	61 (27.4)	48 (19.9)	48 (18.8)	
Hispanic	72 (32.3)	93 (38.6)	65 (25.4)	
Other	16 (7.2)	37 (15.4)	65 (25.4)	
**Parent/caregiver age, mean (SD), y**	36.9 (8.4)	37.0 (10.6)	36.6 (10.9)	.32
**Parent/caregiver BMI (n = 653)^d^, mean (SD)**	25.7 (4.6)	26.2 (5.2)	26.0 (5.2)	.90
**Parent/caregiver race/ethnicity (n = 726)^d^, n (%)**				
White	50 (22.0)	42 (17.1)	55 (21.7)	.005
Black	27 (11.9)	31 (12.7)	35 (13.8)	
Asian	61 (26.9)	53 (21.6)	51 (20.1)	
Hispanic	75 (33.0)	100 (40.8)	74 (29.1)	
Other	14 (6.2)	19 (7.8)	39 (15.4)	
**Purchased children’s meal, n (%) **	124 (48.4)	88 (35.8)	123 (47.3)	.01
**Aware of ordinance (n = 700)^d^, n (%)**	109 (45.2)	70 (29.4)	39 (17.7)	<.001
**Frequency of eating at fast food restaurant (n = 758)^d^, mean (SD), n per month**	5.4 (5.5)	4.5 (4.8)	4.8 (5.2)	.02

Abbreviations: BMI, body mass index; SD, standard deviation.

a The Healthy Food Incentives Ordinance (No. 290–10) was enacted on December 1, 2011.

b Determined by χ^2^ test for difference in proportions across time points.

c Not reported for children younger than 3 years (23). BMI values (kg/m^2^) calculated on the basis of self-reported height and weight; BMI categorized according to age- and sex-specific percentiles.

d Not all survey responses reflect the full sample size because parents/caregivers either declined to answer or (more often) did not know answer to question.

Other exploratory factors potentially affected purchases and toy-related behaviors. Awareness of the ordinance among dyads was moderately high at pre-ordinance time point 1 (45.2%) and decreased thereafter (29.4% at time point 2 and 17.7% at post-ordinance, *P* < .001) (Table 1). The mean frequency of eating at fast food restaurants varied significantly across time (pre-ordinance time point 1, 5.4 times per month; pre-ordinance time point 2, 4.5 times per month; post-ordinance, 4.8 times/month; *P* = .02). Caregivers reported “taste” as a factor influencing purchase of a children’s meal less often over time (*P* = .06) and “child-sized portions” more often over time (*P* < .001) (Table 2).

**Table 2:** Self-Reported Factors That Influence Purchase of Children’s Meal Before and After Enactment^a^ of Toy Ordinance in Fast-Food Restaurants, San Francisco, 2011–2012^b^

Item	Pre-Ordinance Time Point 1 (Jan–Mar 2011)	Pre-Ordinance Time Point 2 (Oct–Nov 2011)	Post-Ordinance (Jan–Mar 2012)	*P* Value^c^
**Factors influencing purchase among children who purchased children’s meal (n = 324)^d^ n (%)**				
Toy	51 (41.1)	40 (46.5)	42 (36.8)	.39
Food my kid likes/taste	38 (30.7)	28 (32.6)	22 (19.3)	.06
Kid portion sizes	18 (14.5)	19 (22.1)	43 (37.7)	<.001
Healthier than adult meal	2 (1.6)	4 (4.7)	2 (1.8)	.37
Always order it	13 (10.5)	0	12 (10.5)	.007
**Frequency of child requests for toys among children who request toys (n = 535), n (%)**				
More than half the time	104 (58.1)	103 (58.9)	117 (64.6)	.06
Half the time	43 (24.0)	26 (14.9)	33 (18.2)	
Less than half the time	32 (17.9)	46 (26.3)	31 (17.1)	

a The Healthy Food Incentives Ordinance (No. 290–10) was enacted on December 1, 2011.

b For all food/beverage purchases (not only children’s meals) among children aged 0–12 years (n = 762).

c Determined by χ^2^ test for difference in proportions across time points.

d Does not reflect the full sample size because parents/caregivers either declined to answer or (more often) did not know answer to question.

Because both restaurant chains responded the same way to the toy ordinance — by charging an additional 10 cents for a toy but not by changing menus to meet ordinance criteria — data were aggregated across restaurants in the primary analyses. The difference in calories per order over time for all children’s orders was not significant (*P* = .20) (Table 3). However, we found a significant decrease in calories per order over time for children’s meals only (*P* < .001). Further analysis (Figure) showed a significant decrease from pre-ordinance time point 1 to pre-ordinance time point 2 and from pre-ordinance time point 1 to post-ordinance (both *P* < .001) but no significant difference between pre-ordinance time point 2 and post-ordinance (*P* = .82).

**Table 3:** Total Calories Per Order Before and After Enactment^a^ of Toy Ordinance, All Children’s Orders (N = 762) and Children’s Meals Only (n = 335), San Francisco, 2011–2012

Item	Pre-Ordinance Time Point 1 (Jan–Mar 2011)	Pre-Ordinance Time Point 2 (Oct–Nov 2011)^b^	Post-Ordinance (Jan–Mar 2012)	*P *Value^b^
**All children’s orders, n **	256	246	260	—
Mean (SD)	686 (337)	662 (351)	654 (318)	
Median (IQR)	610 (515–830)	570 (405–859)	570 (409–786)	.20
**Children’s meals only, n**	124	88	123	—
Mean (SD)	586 (163)	533 (170)	530 (188)	
Median (IQR)	555 (520–642)	515 (409–625)	515 (405–625)	<.001

Abbreviations: —, does not apply; SD, standard deviation; IQR, interquartile range.

a The Healthy Food Incentives Ordinance (No. 290–10) was enacted on December 1, 2011.

b Kruskal–Wallis test used to determine *P *values across time points.

**Figure:**
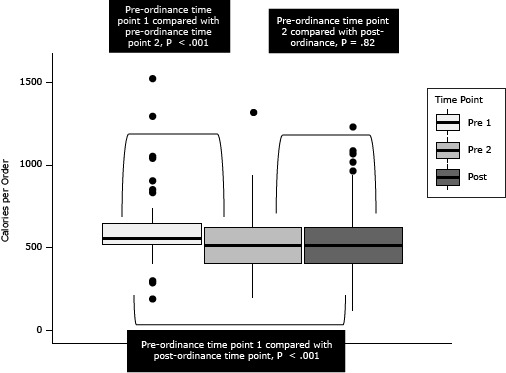
Total calories per order (n = 335) before and after enactment of the San Francisco Healthy Food Incentives ordinance, for children ordering a children’s meal at 2 national restaurant chains in San Francisco, 2011–2012. Mann–Whitney *U* tests were used to test for comparisons between time points. The horizontal line in the middle of each box indicates the median, and the top and bottom borders of the box mark the 75th and 25th percentiles, respectively. The upper whisker extends to the largest data point within 1.5 IQR of the upper quartile while the lower quartile extends to the smallest data point within 1.5 IQR of the lower quartile. Points beyond the whiskers are shown as dots. Abbreviations: Pre 1, pre-ordinance time point 1; Pre 2, pre-ordinance time point 2; Post, post-ordinance time point. Abbreviation: IQR, interquartile range. **Table:** 

Time Point	No. of Calories							
	N (Median)	Interquartile Range	10th Percentile	90th Percentile	Top Whisker	Bottom Whisker	Top Outliers	Bottom Outliers
Pre-ordinance time point 1	124 (555)	520–642	481.5	687	740	395	830, 850, 900, 1040, 1050, 1290, 1520	190, 190, 290, 290, 300
Pre-ordinance time point 2	88 (515)	409–625	390	747	940	190	1320	None
Post-ordinance	123 (515)	405–625	325	781	950	110	960, 1015, 1065, 1085, 1230	None

Because of significant main effects, children’s meal–only orders were disaggregated first by restaurant chain and then by entrée, side dish, beverage, and dessert and tested for differences (Table 4). Post-hoc analyses showed a significant decrease in calories per order over time for Chain A (*P* < .001) but no change for Chain B (*P* = .43). Further analysis of Chain A entrées, side dishes, and beverages showed no difference over time in calories, total fat, or sodium for children’s entrées and no difference in calories or sodium for beverages. However, we found a significant decrease over time in calories, total fat, and sodium for side dishes and in total fat for beverages (due to a substitution of a nonfat beverage for a low-fat beverage).

**Table 4:** Caloric and Nutritional Content of Orders for Children’s Meal Only Before and After Enactment^a^ of Toy Ordinance, by Restaurant Chain, Entrée, Side Dish, and Beverage, San Francisco, 2011–2012^b^

	Pre-Ordinance Time Point 1 (Jan–Mar 2011)	Pre-Ordinance Time Point 2 (Oct–Nov 2011)	Post-Ordinance (Jan–Mar 2012)	*P *Value^c^
**Chain A, kcal/order (n = 243 orders)**	540 (520–642)	435 (405–540)	475 (405–569)	<.001
**Chain B, kcal/order (n = 92 orders)**	598 (510–631)	610 (518–650)	540 (530–700)	.43
**Chain A entrée**				
kcal/entrée	190 (190–280)	190 (190–280)	250 (190–300)	.69
Fat/entrée, g	12 (12–12)	12 (12–12)	12 (12–12)	.68
Sodium/entrée, mg	360 (360–540)	360 (360–540)	520 (360–750)	.63
**Chain A side dish**				
kcal/side	230 (230–230)	115 (100–115)	115 (115–115)	<.001
Fat/side, g	11 (11–11)	5 (5–5)	5 (5–5)	<.001
Sodium/side, mg	160 (160–160)	70 (70–70)	70 (70–70)	<.001
**Chain A beverage**				
kcal/beverage	110 (100–150)	110 (100–130)	110 (100–150)	.81
Fat/beverage, g	0 (0–2)	0 (0–0)	0 (0–0)	.05
Sodium/beverage, mg	15 (5–125)	15 (5–40)	15 (5–125)	.86
**Chain B entrée**				
kcal/entrée	290 (190–300)	260 (190–290)	260 (190–300)	.44
Fat/entrée, g	14 (11–14)	11 (11–17)	11 (11–14)	.73
Sodium/entrée, mg	475 (340–710)	460 (310–490)	490 (310–710)	.21
**Chain B side dish^d^**				
kcal/side	220 (220–220)	220 (220–220)	240 (240–240)	<.001
Fat/side, g	11 (11–11)	11 (11–11)	10 (10–10)	<.001
Sodium/side, mg	340 (340–340)	340 (340–340)	330 (330–330)	<.001
**Chain B beverage**				
kcal/beverage	105 (100–140)	105 (105–109)	105 (103–115)	.98
Fat/beverage, g	0 (0–0)	0 (0–0)	0 (0–0)	.35
Sodium/beverage, mg	15 (0–23)	0 (0–23)	15 (3–23)	.24

a The Healthy Food Incentives Ordinance (No. 290–10) was enacted on December 1, 2011.

b All values are median (interquartile range), unless otherwise indicated.

c Kruskal-Wallis test used to determine *P* value across time points.

d Chain B’s nutritional analysis indicated an increase in calories of value-sized french fries by 20 kcal and a decrease in saturated fat by 1 g and in sodium by 10 mg. To the best of our knowledge, these changes occurred in November 2011 near the end of pre-ordinance time point 2 and appeared to cause significant changes over time in the energy and nutrition values for the category Chain B side dish. However, these changes were not large enough to affect the findings on Chain B orders for children’s meal only.

## Discussion

This study examined the impact of the San Francisco Healthy Food Incentives Ordinance on restaurant response and child food and beverage orders in a natural experiment at global Chain A and B restaurants before and after ordinance enactment. Both restaurant chains used the compliance strategy of offering toys for an additional 10 cents with the purchase of a children’s meal, and neither changed their menus to meet ordinance criteria (24). This compliance strategy did not significantly reduce the proportion of children receiving toys with children’s meals. The restaurants were able to comply in this way because of ordinance language which prohibited only the giving away of *free* toys or other incentives with foods, beverages, and meals not meeting nutritional criteria (8). This language allowed toys to be sold separately.

Restaurants in Santa Clara County responded differently to a similar ordinance, which did not contain the word “free” (12). Santa Clara County restaurants changed their toy distribution and marketing practices in positive ways, including improving the marketing of more healthful children’s meals and discontinuing the giveaway of toys with children’s meals not meeting ordinance criteria or altogether (12). Ordinance wording is a critical element to the outcomes of public health policies (25).

We found a significant decrease in calories per order for children purchasing children’s meals just before ordinance enactment. This decrease appeared to result from menu changes at Chain A from a less healthful to a more healthful default side dish. Additionally, because of the more healthful default side dish and the substitution of a fat-free beverage for a low-fat beverage, we found a significant decrease in total fat and sodium over time in children’s-meal–only orders. These results support research suggesting that more healthful default options may be a powerful approach for improving individual behavior (26).

This study contributes to the limited research on the effects of public health policies aimed at improving restaurant food environments. Strengths of the study are a quasi-experimental design in which restaurants were surveyed before and after enactment of an ordinance at two global fast food chains; the real-world setting; the use of multiple methods of evaluation; and the seasonally matched time points. Additionally, San Francisco has a racially/ethnically diverse population and a large proportion of overweight children. Limitations of the study include the use of self-reported data on food and beverages, height, weight, and other variables that are sensitive to bias (27). Measuring height and weight was not feasible in this setting. Although self-report of food and beverage purchases may introduce response bias, the use of receipts can introduce other inaccuracies or uncertainty. To reduce bias, interviewers were trained to systematically collect food and beverage data, and collection was monitored weekly. Another limitation was the short duration of the study (1 year) and small sample size. The street-intercept method also may have introduced response bias; those willing to be surveyed may have differed from those not willing. Finally, the data are limited to San Francisco, and the external validity of the findings is not known.

This study illustrates two major findings: one, the wording of regulations and policies can have significant impacts on compliance, and two, more healthful default menu options can have a rapid and positive impact on the healthfulness of food and beverage purchases. Moreover, although Chain A menu changes may not have been caused by the ordinance, the transition to a more healthful default side dish and fat-free beverage option were consistent with the ordinance intent. Finally, a valuable aspect of these types of changes is their potential reach: Chain A’s transition to a more healthful default offering, which began in September 2011 only in California, was reported by Chain A to be diffused to all US restaurants by the spring of 2012 (20).
